# Resident Peer Review Process: An Innovative Teaching Model in an Internal Medicine Residency Program

**DOI:** 10.7759/cureus.14017

**Published:** 2021-03-21

**Authors:** Srikrishna V Malayala, Khalid Qazi, Deepa Vasireddy, Paavani Atluri

**Affiliations:** 1 Internal Medicine, Temple University Hospital, Philadelphia, USA; 2 Internal Medicine, State University of New York (SUNY) Buffalo, Buffalo, USA; 3 Pediatrics, Pediatric Group of Acadiana, Lafayette, USA; 4 Internal Medicine, Bay Area Hospital, Coos Bay, USA

**Keywords:** peer reviews, gme scholarly activity, medical residency, teaching technology, internal medicine residency, acgme core competencies, patient safety, quality improvement and patient safety

## Abstract

Introduction

Medical peer review is an integral part of the performance improvement process in any hospital. The very first exposure to the peer review process in a physician’s career is typically after graduation and when they start practicing as an attending physician. We implemented a “Resident Peer Review Process” training module in our Internal Medicine residency program with an intention to familiarize the residents about peer review.

Methods

The resident peer review process was implemented over a period of four weeks with the “resident peer review committee” having representation from all three Postgraduate Year (PGY) levels. The committee reviews the cases referred to them over a period of four weeks through a process of weekly meetings and adjudicates the cases. If any deficiency is identified, the committee will provide feedback to the residents involved in the case and presents the educational points identified from the adjudication at the end of the module as a morning report.

Results

Eighty-nine (89) cases were reviewed through this process over a span of two years. About 77.5% of the cases were identified to have a deficiency. Teaching points were identified and presented in Week 4 meetings in 80.9% of the cases that had a deficiency. The residents provided a positive response and said that the process improved their quality of patient care (98%), professionalism (95%), systems-based practice, practice-based learning (90%), medical knowledge (88%), and interpersonal and communications skills (87%).

Discussion

This resident-driven, novel, and innovative model can be a successful teaching methodology for Internal Medicine residents to augment Patient Safety and Quality Improvement and could be implemented in residency programs irrespective of the size and specialty.

## Introduction

Medical peer review is the process by which a committee of physicians examines the work of a peer and determines whether the physician has met accepted standards of care [1]. It has been an integral part of patient safety and quality improvement in all hospitals.

In 2007, the Accreditation Council for Graduate Medical Education (ACGME) Committee on Innovation called for quality improvement initiatives in teaching institutions. ACGME identified that the peer review process implements “Practice-Based Learning and Improvement” and recommended peer review for evaluating the residents [2].

Typically, the first exposure to the medical peer review process is only when the residents graduate and start practicing at the level of an attending physician. Without an orientation to the peer review process, the physicians might not be able to appreciate the significance of peer review during their early years of practice and might even consider it punitive instead of welcoming it as a quality or performance improvement process. With an intention to familiarize the residents with the peer review process, we implemented the “Resident Peer Review Process” and incorporated it into the Performance Improvement curriculum in our Internal Medicine residency program.

## Materials and methods

Our Internal Medicine residency program is a moderate-sized training program based out of two community hospitals in a major metro city in Western New York, USA [3]. The program has a total of 37 medical residents every year. Since 2006, the program has had a four-week rotation titled ‘Performance Improvement Rotation’ and focused on training the residents in five specific areas: Core measures, the departmental peer review process, clinical documentation improvement, patient safety goals, and adverse event reporting. This four-week rotation was designed as a mandatory module for the second-year Internal Medicine residents [4].

The Resident Peer Review process

With our resident peer review process, we developed a curriculum for each level of training. The entire process was run over four weeks (for one entire module). In every module, there would be a different “resident peer review committee” so that all the residents get exposure to the process of being reviewed by their peers at the end of the study.

After successfully implementing the rotation and running it over a span of two years, we decided to analyze the rotation by taking feedback from the residents who were part of this process. A 28-item tool (Likert scale: range 1-5; with 4-5 considered positive response) was administered to the residents. All the archived data from 23 peer review cycles (23 modules) over a period of two years were reviewed and analyzed.

## Results

A total of 39 residents from all three PGY levels reviewed the rotation. The residents provided a positive response and said that it is, in fact, improving their quality of patient care (98%), professionalism (95%), systems-based practice (SBP) and practice-based learning and improvement (PBLI; 90%), medical knowledge (88%), and ICS (87%) core competencies. 

Ninety-two point five percent (92.5%) of the residents felt that the peer review process was beneficial for their professional development, with a score of 4.49/5 on the Likert scale. Eighty-nine point eight percent (89.8%) of the residents felt that the peer review process was well-structured (4.28/5). Eighty percent (80%) reported that the peer review process was conducted in the most professional way (4.22/5). Ninety-five percent (95%) agreed that the peer review process is not punitive towards residents and the intent is educational (4.53/5). Ninety-two point five percent (92.5%) of them felt that faculty supervision was supportive and not dictatorial (4.43/5). All 100% of the residents felt that the 'Outcomes from Peer Review' morning report was educational (Table 1).

**Table 1:** Evaluation of the rotation by the Internal Medicine residents

Evaluation question	Avg score (1-5)	Positive Response (%)
The peer review process was beneficial to me for my professional development	4.49	92.5%
Peer review process was well-structured	4.28	89.8%
Peer review process was conducted in the most professional way	4.22	80%
Peer review process is not punitive towards residents and the intent is educational	4.53	95%
Faculty supervision was supportive and not dictatorial	4.43	92.5%
The 'Outcomes from Peer Review' morning report is educational	4.53	100%
Resident Peer Review is beneficial to ensure patient safety	4.41	87.2%
Resident Peer Review is beneficial for resident education	4.47	90%

## Discussion

The intention of this rotation is to orient the internal medicine residents to the concept of patient safety and quality improvement. In any institution, peer review is an essential and key component of patient safety. After implementing the rotation for almost two years, all the residents who experienced this process have given favorable reviews. With this, we decided to continue the resident peer review process as a requirement in our teaching program.

With this manuscript, we also intend to describe the overall rotation in detail.

Description of the rotation

**Figure 1:**
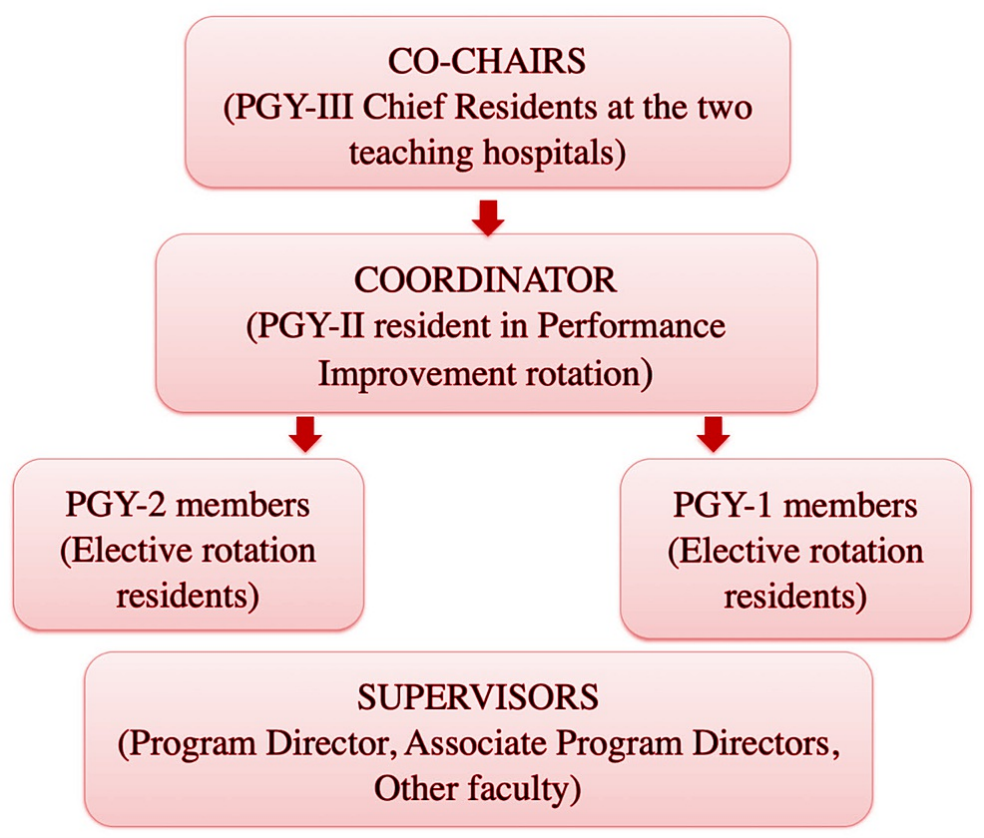
Composition of the Resident Peer Review committee

It was ensured that the committee had representation from all three Postgraduate Year (PGY) levels (Figure 1). 

From PGY-3, the two chief residents were part of the committee. The two chief residents are named the “Co-chairs” of the committee. From PGY-2, the resident rotating in the Performance Improvement (PI) rotation and those on elective rotations like ambulatory medicine, research block, pathology, stress lab, anesthesia, radiology, nuclear medicine, etc., are part of the committee. The PI resident is automatically designated as the “coordinator” of the committee. From PGY-1, the interns on elective rotations were part of the committee. The program director (PD) and associate program directors (APD) supervised the peer review process.

The committee members meet every Friday morning as part of the morning report. Cases to review were referred to the co-chairs from either the program director, associate program directors, other faculty, other residents, and sometimes the nursing, pharmacy, and other departments.

Roles and responsibilities of committee members

The responsibilities of the Co-chairs (chief residents) include chairing the meetings, assigning charts for review, guiding and supervising the co-ordinator, being a resource to the team members, and being a liaison to the program director.

Responsibilities of the coordinator (PGY-2 resident on PI rotation) include collecting charts and forwarding them to the reviewers, arranging meeting dates and rooms, informing/emailing all concerned about the meetings, serving as a liaison between the co-chairs and committee members, maintaining minutes of the committee, and being a resource for the team members.

The responsibility of the other residents from the PGY-2 and PGY-1 years was to perform a literature review of the involved cases, perform a chart review of the cases as assigned by the co-chairs and coordinator, identify the deficiencies and/or opportunities for improvement, and prepare a presentation of the case for the weekly meeting. The PGY-1 residents also present the cases in the Week 4 meeting.

Structure and process of the Resident Peer review process

**Figure 2:**
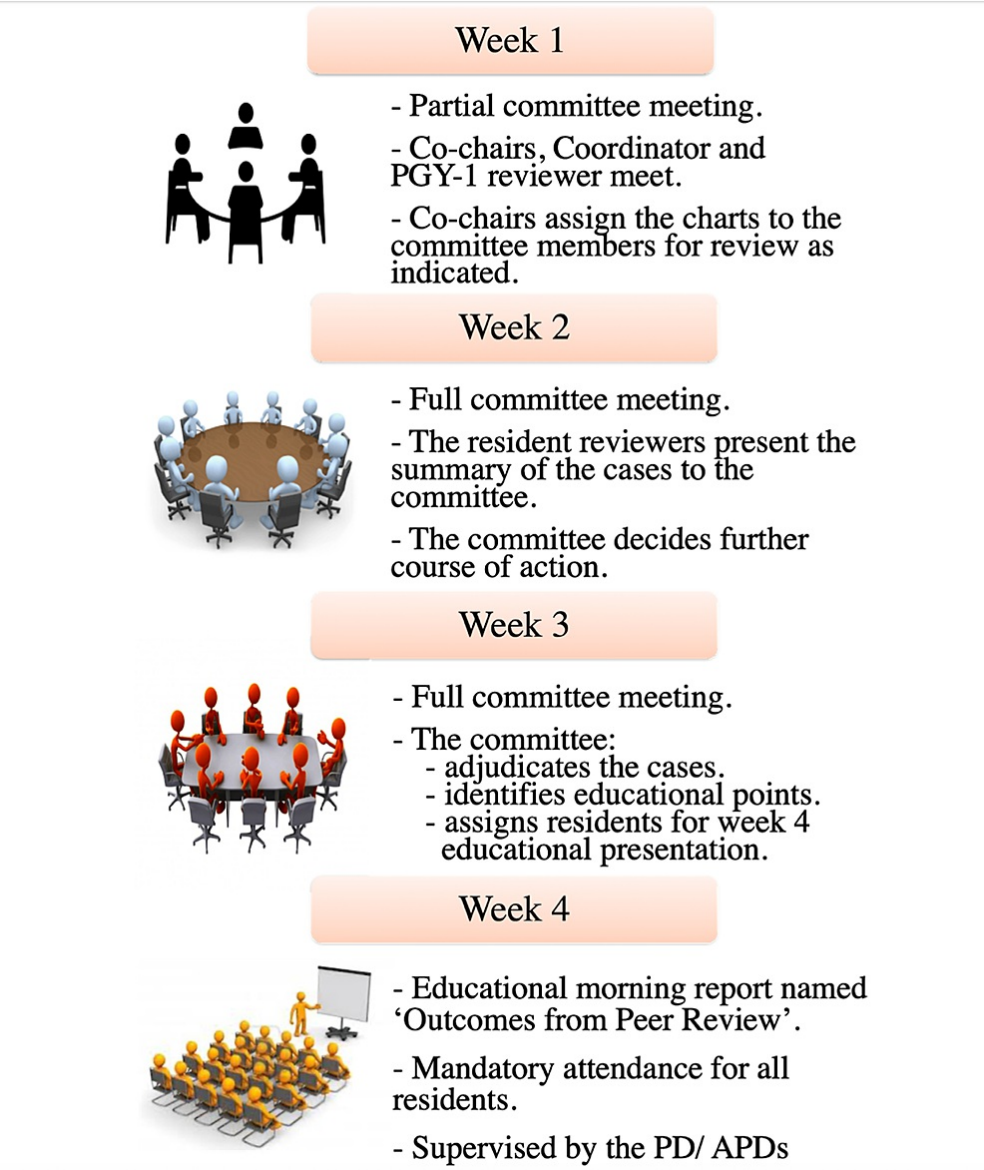
The four-week Resident peer review cycle

This process is depicted in Figure 2.

The one-month cycle of the peer review process was conducted as follows:

Week 1: The meeting on the first Friday morning of the module is a partial committee meeting. The Co-chairs, coordinator, and PGY-1 reviewers meet during the first week for chart assignment and defining roles to the committee members.

Week 2: This meeting, on the morning of the second Friday is a full committee meeting. The co-chairs looked at the progress of chart review and sought initial reports from the reviewers. The committee decides the further course of action. The committee decides if the residents involved in the review case should be called so that they have at least a week to review the chart.

Week 3: This is a full committee meeting. The whole committee meets and adjudicates the cases one by one and decides on a disposition. The committee also identifies the educational points and plans for the educational presentation for the Week 4 meeting.

Week 4: This is an educational “morning report” named ‘Outcomes from Peer Review.’ As this meeting is a morning report, it required mandatory attendance from all residents and was supervised by the PD/APDs.

Members from other departments like case management, pharmacy, and PI were sometimes invited to join Week 3 and Week 4 meetings and their input was taken to decide the appropriate disposition or deliver a didactic lecture.

We also performed an evaluation of the overall outcomes in terms of patient safety itself. In this span of two years, a total number of 89 cases were reviewed. Out of the 89 cases reviewed, 20 (22.5%) cases revealed no deficiencies. In these 69 cases that were identified as deficient, 7% of the cases were identified to have a “severe deficiency” and the residents involved in those cases were called in for one-to-one counseling by either the chief residents or the program director/associate program directors. Teaching points were identified and presented in the Week 4 meetings in 80.9% of the cases.

The core competencies found deficient were SBP in 47% of the cases, patient care in 45%, medical knowledge in 38%, interpersonal and communication skills (ICS) in 36%, PBLI in 32%, and professionalism in 26% (Table 2) [5].

**Table 2:** Outcome of the cases reviewed in the peer review process and their adjudication PGY: postgraduate year; ICU: intensive care unit; CCU: critical care unit

Total cases reviewed (n=89 cases)	
Cases identified with a deficiency	69 (77.5%)
Cases identified with a severe deficiency	7 (7.8%)
Cases identified without any deficiency	20 (22.5%)
Core competency identified as a deficiency (n=69 cases)	
Systems-based practice	47%
Patient care	45%
Medical knowledge	38%
Interpersonal and communication skills	36%
Practice-based learning and improvement	32%
Professionalism	26%
Resident deficiency based on their level of training (n=69 cases)	
PGY-1	23.3%
PGY-2	61.8%
PGY-3	16.9%
Type of service	
General medicine floor rotation	36.0%
Critical care rotation (ICU/CCU)	34.8%
Night float rotation	27.0%
Day float (day admitting) rotation	2.2%

The PGY-2 residents (senior residents) were more often identified as having a deficiency after reviewing these cases (61.8%). In 16.9% of the cases, the PGY-3 residents were identified as deficient, and in 23.3% of the cases, the resident was identified as a PGY-1 (Table 2).

About 36% of the cases were from the general medicine floor rotations, 34.8% of the cases were from critical care rotations, 27% from night float, and the rest 2.2% cases were from the day float (day admitting) rotation.

## Conclusions

We believe that this resident-driven, novel, and innovative model can be a successful teaching methodology for Internal Medicine Residents to augment Patient Safety and Quality Improvement as well as to effectively address learning in terms of core competencies. The rotation has received an overwhelmingly positive response from our residents, and we intend to continue the process in our residency program. We strongly encourage that such a module should be implemented in all the training programs irrespective of the specialty and size of the program.
